# Analysis of ventilator induced lung injury impact in lung and cardiac tissue in a murine model

**DOI:** 10.1186/2197-425X-3-S1-A565

**Published:** 2015-10-01

**Authors:** E Correger, J Marcos, DE Sotelo, M Beldarrain, P Stringa, G Laguens, J Lofeudo, L Vittone

**Affiliations:** Medical Sciences Faculty, Universidad Nacional de La Plata, La Plata, Argentina; Hospital El Cruce, UTIA, Florencio Varela, Argentina; HIGA San Martín La Plata, Rheumatology Unit, La Plata, Argentina; Hospital Santamarina, UTI, Tandil, Argentina; Fundación Favaloro, Trasplante, Buenos Aires, Argentina; Universidad Nacional de La Plata, Cátedra Patología, La Plata, Argentina; Universidad Nacional de La Plata, CIC, La Plata, Argentina; Universidad Nacional de La Plata, Cátedra de Fisiología, La Plata, Argentina

## Introduction

Mechanical ventilation (MV) with high tidal volumes (Vt) leads to enormous biophysical lung damage, both in patients and in experimental animal models. From a biological point of view, however, the inflammatory response triggered by high Vt-MV and its association with the development of multisystem organ failure is still under study.

## Objective

Based on an acute model (3 hr) of MV with high Vt in rats, we intend to characterize the clinical and histopathological pattern of cardiac injury resulting from injurious MV.

## Methods

8 male Wistar rats were mechanically ventilated during an initial stabilization period of 20 min with Vt = 8 ml/kg, PEEP = 5 cmH2O and FiO2 = 0.4, followed by Vt = 25 ml/kg and ZEEP for 3 hr (HVt group). A sham group (n = 6) was maintained with spontaneous ventilation during the same period. Arterial blood gases, mean arterial pressure (MAP) and lung weight gain were measured. Lung injury (ATS score) and pathology of the apex was evaluated histologically. The data were analysed by t-test (* p < 0.05).

## Results

No statistically significant changes were observed in hemodynamic and respiratory oxygenation between groups. The HVt group showed pulmonary oedema, worsening of the pulmonary histological consisting in benign vascular damage, and myocyte injury. (Table 1)

**Table 1:** Results

	SHAM n = 6	HVt n = 6
MAP	95 ± 5	89 ± 7
PaO2/FiO2	225 ± 26	280 ± 13
Weight gain (g)	0.43 ± 0.017	0.92 ± 0.59*
Pulmonary histology	0.0050 ± 0.0077	0.030 ± 0.003*
Myocardium histology	3.16 ± 2.8	8.3 ± 0.9*

## Conclusions

Under these experimental conditions, animals ventilated with high Vt had pulmonary oedema and worsening of ATS score, associated to cardiac structural changes compatible with myositis and necrosis.

